# Analysis of the risk factors of the radiation-induced encephalopathy in nasopharyngeal carcinoma: A retrospective cohort study

**DOI:** 10.1515/med-2024-0911

**Published:** 2024-08-20

**Authors:** Xiong Ran, Shaoquan Zhou, Kang Li, Shijun Qiu, Yikai Xu, Min Xu, Ruimeng Yang

**Affiliations:** Department of Radiology, Chongqing General Hospital, Chongqing, 401147, China; Department of Medical Imaging Center, Nan Fang Hospital, Southern Medical University, No. 1838, North Guangzhou Avenue, Baiyun District, Guangzhou, 510515, China; Department of Medical Imaging Center, Nan Fang Hospital, Southern Medical University, Guangzhou, 510515, China

**Keywords:** radiation-induced encephalopathy, nasopharyngeal carcinoma, risk factors, retrospective cohort study, medical imaging examination

## Abstract

To analyze the related factors of radiation-induced encephalopathy in nasopharyngeal carcinoma (NPC) to identify the risk factors and their clinical significance. This retrospective cohort study included 707 NPC patients. They had undergone conventional and enhanced computed tomography or magnetic resonance imaging scans. They were divided into the radiation-induced encephalopathy group and the no encephalopathy group according to the imaging examination. Detailed clinical information was collected. The incidence of radiation-induced encephalopathy in NPC was 22.2%, in which 124 were radiation-induced encephalopathy and 33 were reirradiation patients. We found that age, pathological type, radiation method, hypertension, radiation course, relapse, carotid/cerebral arteriosclerosis, clinical stage, and radiotherapy dose were statistically significant between the two groups (*p* < 0.05). Multiple logistic regression showed that clinical stage, age, radiotherapy method, hypertension, carotid/cerebral arteriosclerosis, and radiation courses after a reoccurrence of NPC were risk factors for radiation-induced encephalopathy. The more advanced the clinical stage was and the older the patient, the greater the risk. Radiotherapy method, radiation course, hypertension, carotid/cerebral arteriosclerosis, age, and clinical stage were the risk factors associated with radiation-induced encephalopathy in NPC.

## Graphical abstract

**Figure:**
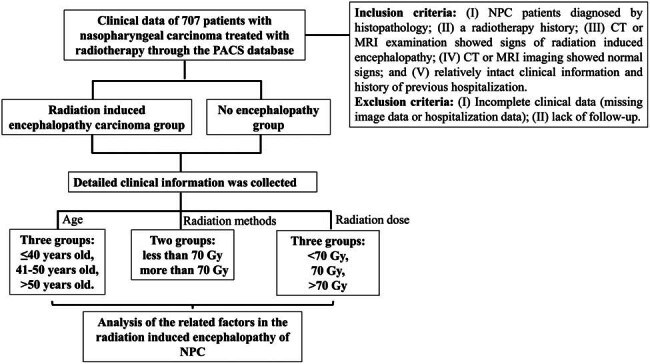

## 1 Introduction

After receiving radiotherapy, patients will suffer varying degrees of radiotherapy-induced brain injury, and a portion of them may develop radiation-induced encephalopathy, which can seriously deteriorate their quality of life. Many subtypes of brain tumors unfortunately have a suboptimal long-term clinical outcome, especially in children [1]. The incidence rate of radiation-induced encephalopathy after radiotherapy for patients with nasopharyngeal carcinoma (NPC) ranges from 0.9 to 4% [2]. Cai reported an incidence rate of 19.5% from a review of 5 years of follow-ups in the picture archiving and communication system (PACS) database [3]. In the past, patients who underwent head computed tomography (CT) and magnetic resonance imaging (MRI) scans after neurological symptoms have appeared and are often prone to misdiagnosis. Therefore, the actual incidence rate may be higher.

Ionizing radiation and a history of allergies are the only well-validated risk factors for brain tumors [4]. A retrospective study of 168,394 children found that those children who received one or more CT scans between 1979 and 2012 had a significantly higher incidence of brain tumors than the general population [5]. The risk of brain tumors may be increased by a relatively high brain dose of head CT scans (20–50 mGy) [5].

Clinical outcomes and prognosis are not uniform due to individual patient differences and different treatment approaches. In previous studies, the number of radiation-induced encephalopathy for NPC was too small to identify the risk factors [2–5]. Rare studies on radiation-induced encephalopathy for NPC can be searched for. Therefore, it is critical to explore the incidence rate and risk factors of radiation-induced encephalopathy of NPC. In this study, we investigated the relationship between the incidence and associated risk factors based on the analysis of factors associated with radiation-induced encephalopathy in NPC. Therefore, while clarifying the relevant influencing factors, it is of great significance to give corresponding protective measures to reduce the incidence of radiation-induced encephalopathy in NPC patients.

## 2 Subjects and methods

### 2.1 Subjects

A total of 707 NPC patients treated with radiotherapy through the PACS database in the Southern Hospital between December 2002 and December 2010 were included. They had undergone conventional and enhanced CT or MRI scans. Detailed clinical information was collected for further analysis. According to the patient’s desire for treatment, patients were divided into the radiation-induced encephalopathy carcinoma group and the no encephalopathy group.

### 2.2 Diagnoses of radiation-induced encephalopathy

Radiation-induced encephalopathy of NPC is the brain parenchymal injury and nerve damage caused by radiotherapy in NPC patients, which can affect the nervous system functions, leading to dizziness, headache, mental disorders, motor dysfunction, and other symptoms. If not timely intervened, it can lead to irreversible neurological damage, which needs to be diagnosed and treated as soon as possible to avoid aggravating the neurological damage and increasing the risk of poor prognosis. The diagnosis of radiation-induced encephalopathy in NPC was made by a history of radiotherapy and related imaging examination, and all patients underwent CT and/or MRI examination of the head. First, the NPC patients had a history of radiotherapy, which is the most important treatment history. Then, the bilateral temporal lobes were mostly involved in radioactive encephalopathy of NPC, while the unilateral temporal lobe was relatively rare. Radiation-induced encephalopathy can be roughly divided into edematous necrosis type and liquefied cyst type by CT plain scan. The edematous necrosis type was more common, mainly with large low-density shadows at the lesion site and unclear boundaries. CT scan of the liquefied sac showed a hypodense shadow with clear boundaries and no significant surrounding edema. MR Plain scan showed a low signal on T1WI, a high signal on T2WI, and a high signal on fluid-attenuated inversion recovery, and the enhanced scan showed garland or gyri enhancement. We should exclude this invasion of the base of the skull, brain metastasis, or brain tumors, and no history of cerebrovascular disease or intracranial surgery.

### 2.3 Selection criteria

Inclusion criteria: (1) NPC patients diagnosed by histopathology; (2) a history of radiotherapy; (3) CT or MRI examination showed signs of radiation-induced encephalopathy; (4) CT or MRI imaging showed no clear signs of invasion of the base of the skull, brain metastasis, or brain tumors, and no history of cerebrovascular disease or intracranial surgery, and (5) relatively intact clinical information and history of previous hospitalization. Exclusion criteria: (1) incomplete clinical data (missing image data or hospitalization data) and (2) lack of follow-up.

### 2.4 Analysis of related factors

To analyze the related factors in the radiation-induced encephalopathy of NPC, detailed clinical information was collected, including gender, age, pathological type, clinical stage, lymph node condition of the neck, metastasis, radiation method, radiation dose, chemotherapy, hypertension, diabetes mellitus, arteriosclerosis of the cerebral and carotid arteries, other underlying diseases, relapse, radiation courses, etc.

Age: The majority of patients were 40–50 years old, and the number of patients over 50 years were less. Therefore, in order to ensure that the number of patients in each group was roughly the same, the patients were divided into three groups: ≤40 years old, 41–50 years old, and >50 years old.

Radiation methods: Patients were grouped into conventional external radiotherapy and intra-cavitary radiotherapy, three-dimensional conformal radiotherapy, and intensity-modulated radiation therapy according to the radiation methods. At that time, 70 Gy was widely used for radical radiotherapy of NPC in China. A small number of patients received radiation dose less than 70 Gy or more than 70 Gy. Therefore, grouping by different radiation doses can compare the effects of different radiation doses on the occurrence of radiation-induced encephalopathy in NPC.

Radiation dose: For radiotherapy grouping, patients were placed in three groups according to the radiation dose: <70, 70, and >70 Gy.

### 2.5 Sample size estimation

This study required more data to get more accurate results. It was estimated that at least 500 cases were needed. Due to the high incidence of NPC in Guangdong Province, our hospital has a large number of NPC patients and post-treatment reexamination patients.

### 2.6 Statistical analysis

SPSS 26.0 software (IBM Corp.) was used to conduct the analyses. All measurement data were expressed as mean ± standard deviation. In the univariate analysis for the radiation-induced encephalopathy of NPC, the comparison of the counting data was performed with the Pearson chi-squared test, while data with a non-normal distribution were compared with the Wilcoxon test. According to the univariate analysis, we determined whether the occurrence of radiation-induced encephalopathy was a binary variable. Multiple logistic regression analysis was used to analyze the related factors (*α* = 0.05). *p* < 0.05 was considered to indicate a statistically significant difference.


**Ethical approval:** The authors are accountable for all aspects of the work in ensuring that questions related to the accuracy or integrity of any part of the work are appropriately investigated and resolved. The study was conducted following the Declaration of Helsinki (as revised in 2013). The study was approved by the institutional/regional/national ethics/committee/ethics board of Nan Fang Hospital, Southern Medical University (No. XJS S2021-106-01).
**Informed consent:** Informed consent was taken from all the patients.

## 3 Results

### 3.1 General clinical information

Of the 707 patients, 516 were male and 191 were female. They were aged 13–77 years, with a median age of 45.9 years. The patients were divided into the radiation-induced encephalopathy group (*n* = 157) and the no encephalopathy group (*n* = 550). The incidence of radiation-induced encephalopathy in NPC was about 22.2%.

### 3.2 Latent period of radiation-induced encephalopathy in NPC

There were 157 NPC cases of radiation-induced encephalopathy, among which 124 were radiation-induced encephalopathy and 33 were reirradiation. The primary radiotherapy latent period was 48.27 ± 39.97 months, while that of reirradiation was 46.18 ± 35.24 months (*Z* = −0.246; *p* = 0.806, Table 1).

**Table 1:** Radiation-induced encephalopathy latent period of the patients who underwent primary radiotherapy or reirradiation

Radiation course	*n*	Radiation encephalopathy latent period	Median	Min	Max	*Z*	*p*
Primary radiotherapy	124	48.27 ± 39.974	36	1	240	−0.246	0.806
Reirradiation	33	46.18 ± 35.243	36	4	132		

### 3.3 Related factors of the radiation-induced encephalopathy of NPC

#### 3.3.1 Univariate analysis

The analysis of the related factors for radiation-induced encephalopathy showed that gender, multidrug combination chemotherapy, lymph node conditions, diabetes mellitus, and metastasis were not significantly different (*p* > 0.05, Table 2). Meanwhile, age, pathological type, radiation method, hypertension, radiation course, and relapse were statistically significant (*p* < 0.001), as well as carotid/cerebral arteriosclerosis, clinical stage, and radiotherapy dose (*p* < 0.05).

**Table 2:** Univariate analysis of radiation-induced encephalopathy

Factors		No REP (%)	REP (%)	Total (%)	*χ* ^2^/*Z*	*p*
Gender	Female	156 (81.7)	35 (18.3)	191 (100.0)	2.283	0.131
	Male	394 (76.4)	122 (23.6)	516 (100.0)		
Age (years)	≤40	223 (82.9)	46 (17.1)	269 (100.0)	15.466	0.000
	41–50	172 (80.8)	41 (19.2)	213 (100.0)		
	>50	155 (68.9)	70 (31.1)	225 (100.0)		
Pathological type	Poorly differentiated SCC	267 (69.9)	115 (60.1)	382 (100.0)	31.373	0.000
	Well-differentiated SCC	4 (80.0)	1 (20.0)	5 (100.0)		
	Non-keratinization differentiated cancer	48 (88.9)	6 (11.1)	54 (100.0)		
	Non-keratinization undifferentiated cancer	227 (87.3)	33 (12.7)	260 (100.0)		
Clinical stages	Stage I	23 (85.2)	4 (14.8)	27 (100.0)	−2.755	0.006
	Stage II	159 (87.8)	22 (12.2)	181 (100.0)		
	Stage III	182 (79.1)	48 (20.9)	230 (100.0)		
	Stage IVa	95 (72.0)	37 (28.0)	132 (100.0)		
	Stage IVb	65 (81.2)	15 (18.8)	80 (100.0)		
Radiation method	Conventional	205 (67.4)	99 (32.6)	304 (100.0)	31.895	0.000
	Three-dimensional conformal	155 (84.2)	29 (15.8)	184 (100.0)		
	Intensity-modulated radiation therapy	86 (91.5)	8 (8.5)	94 (100.0)		
Combination of multidrug chemotherapy	No	45 (77.6)	13 (22.4)	58 (100.0)	0.136	0.712
	Yes	481 (79.6)	123 (20.4)	604 (100.0)		
Radiation dose (Gy)	<70	58 (90.6)	6 (9.4)	64 (100.0)	12.336	0.002
	70	391 (79.5)	101 (20.5)	492 (100.0)		
	>70	47 (66.2)	24 (33.8)	71 (100.0)		
Lymph node condition	No lymphadenopathy	91 (79.1)	24 (20.9)	115 (100.0)	3.760	0.709
	Left side lymphadenopathy	64 (85.3)	11 (14.7)	75 (100.0)		
	Right side lymphadenopathy	67 (84.8)	12 (15.2)	72 (100.0)		
	Bilateral lymphadenopathy	84 (81.6)	19 (18.4)	103 (100.0)		
	Left neck mass	73 (83.9)	14 (16.1)	86 (100.0)		
	Right neck mass	67 (81.7)	15 (27.3)	82 (100.0)		
	Bilateral neck mass	24 (72.7)	9 (27.3)	33 (100.0)		
Hypertension	No	471 (81.1)	110 (18.9)	581 (100.0)	25.372	0.000
	Yes	39 (84.9)	32 (45.1)	71 (100.0)		
Diabetes mellitus	No	501 (78.4)	138 (21.6)	639 (100.0)	0.802	0.370
	Yes	14 (70.0)	6 (30.0)	20 (100.0)		
Carotid/cerebral arteriosclerosis	No	481 (79.4)	125 (20.6)	606 (100.0)	6.692	0.010
	Yes	29 (63.0)	17 (37.0)	46 (100.0)		
Radiation course	Primary radiotherapy	517 (80.7)	124 (19.3)	641 (100.0)	32.551	0.000
	Reirradiation	33 (50.0)	33 (50.0)	66 (100.0)		
Relapse	No	499 (79.8)	126 (20.2)	625 (100.0)	13.064	0.000
	Yes	51 (62.2)	31 (37.8)	82 (100.0)		
Metastasis	No	468 (78.0)	132 (22.0)	600 (100.0)	0.098	0.754
	Yes	82 (76.6)	25 (23.4)	107 (100.0)		

REP, radiation encephalopathy; SCC, squamous cell carcinoma.

### 3.4 Multiple logistic regression of the radiation-induced encephalopathy

According to univariate analysis (Tables 1–3), we determined whether the occurrence of radiation-induced encephalopathy was a binary variable. Multiple logistic regression of radiation-induced encephalopathy showed that clinical stage, age, radiotherapy method, hypertension, carotid/cerebral arteriosclerosis, and radiation courses after NPC reoccurrence were the risk factors for radiation-induced encephalopathy (Table 3). The odds ratios (Exp[*B*]) of hypertension, carotid/cerebral arteriosclerosis, and reirradiation course were 3.6, 2.883, and 3.94, respectively. The risk ratio of the later clinical stage was 1.279 times greater than that of the previous clinical stage. Similarly, the risk ratio in the older group was 1.417 times greater than that in the lower age group. Conventional and three-dimensional conformal radiation therapy was associated with a risk for radiation-induced encephalopathy of NPC that was 6.202 times and 2.384 times greater than that of intensity-modulated radiation therapy, respectively. Therefore, intensity-modulated radiation therapy may control the occurrence of radiation-induced encephalopathy.

**Table 3:** Multiple logistic regression of radiation-induced encephalopathy

Variable	Regression coefficient (*B*)	Standard error	Wald	*p*	Exp (*B*)	95% CI for Exp (*B*)	
						Lower	Upper
Constant term	−4.593	0.671	46.909	0.000	0.010		
Age	0.348	0.152	5.251	0.022	1.417	1.052	1.908
Clinical stage	0.246	0.111	4.895	0.027	1.279	1.028	1.591
Radiation method							
Conventional radiotherapy	1.825	0.448	16.602	0.000	6.202	2.578	14.92
Three-dimensional conformal radiotherapy	0.869	0.480	3.270	0.071	2.384	0.930	6.114
Hypertension	1.281	0.361	12.603	0.000	3.600	1.775	7.301
Carotid/cerebral arteriosclerosis	1.059	0.408	6.745	0.009	2.883	1.297	6.409
Reirradiation	1.371	0.362	14.366	0.000	3.940	1.939	8.005

## 4 Discussion

### 4.1 Basic situation of radiation-induced encephalopathy in NPC

In our study, the incidence rate of radiation-induced encephalopathy in NPC patients was 22.2%, which was higher than that typically reported in the literature [2,3]. In the past, patients were often prone to misdiagnosis due to the delay in seeking medical attention and backward technology. Therefore, the actual incidence rate may be higher. The latent period of reirradiation is shorter than that of primary radiotherapy. In our results, there was no significant difference between the two groups, with the average latent period of reirradiation being 2 months shorter than that of the primary radiotherapy.

### 4.2 Analysis of the related factors of radiation-induced encephalopathy in NPC patients

The current study showed that age, clinical stage, radiation methods, hypertension, carotid/cerebral arteriosclerosis, and reirradiation of NPC were the risk factors for radiation-induced encephalopathy in NPC patients, with conventional radiation therapy being the riskiest factor.

Due to poor distribution of the radiation dose in conventional radiotherapy in the target area and adjacent tissues, as well as the limited protection of the lead plates of normal tissues, adjacent tissues may receive an excessive radiation dose. In contrast, three-dimensional conformal radiotherapy allows the dose distribution to be visualized in three dimensions according to the shape of the lesion so that the concentration at the lesion site is as high as possible, reducing the radiation dose to the parotid gland, spinal cord, and brainstem. However, this method does not allow for adjustment of the spot dose to the target area as required. Intensity-modulated radiotherapy solves these problems, as it can adjust the radiation dose to the target area based on clinical need [6]. This approach allows for any point of the target area to obtain the ideal dose, with the tumor target area being administered the highest curative dose. Moreover, it can protect the sensitive normal tissues and important organs near the target and protect the sensitive organs embedded within or surrounding the tumor [7,8]. Therefore, intensity-modulated radiation can significantly reduce the incidence of radiation-induced encephalopathy in NPC compared to other radiation therapy methods.

Reirradiation increases the incidence of radiation-induced encephalopathy due to multiple radiation sessions having a cumulative effect on normal tissues, which leads to the reduced tolerance of normal brain tissues. Tang et al. reported that the incidence of primary radiotherapy was 1.3%, while that of reirradiation was 7.1% [9]. Han et al. found that the incidence of primary radiotherapy was 2.07%, while that of reirradiation was 9.6% [10]. In our study, there were 82 recurrent patients, 66 of them underwent reirradiation, so the incidence was 50%, which was significantly higher than that of the primary radiotherapy, and our results were consistent with these above-mentioned studies.

Long-term hypertension causes cerebral artery spasm, stenosis, blood flow obstruction, decreased vascular compliance, arterial insufficiency, ischemia, and hypoxia of brain tissues, which, in turn, lead to the occurrence of radiation-induced encephalopathy [11]. The relevant literature suggests that hypertension is a risk factor for the occurrence of radiation-induced encephalopathy [12,13], but this is only speculation. Some large-scale clinical studies reported that the association between the occurrence of radiation-induced encephalopathy and hypertension is rare. In this study, a close relationship between hypertension and radiation-induced encephalopathy was found. In addition, our study shows that carotid/cerebral atherosclerosis is also a risk factor for the development of radiation-induced encephalopathy. Carotid ultrasound-confirmed carotid atherosclerosis and magnetic resonance angiography-confirmed intracranial atherosclerosis are classified as vascular risk factors.

One study [14] reported that the incidence of carotid and cerebral arteriosclerosis and stenosis after radiotherapy in patients with head and neck cancers was higher than in those who did not receive radiotherapy. Chung et al. [15] observed the change in the common carotid artery and internal and external carotid artery before and after neck radiotherapy for 6–30 months through magnetic resonance angiography. They found that radiotherapy caused premature arteriosclerosis, with the degree of vascular injury being proportional to the radiation dose. NPC patients after radiotherapy often acquire radiodermatitis and neck tissue fibrosis, resulting in carotid vascular degeneration and sclerosis, or pressed vessels via the hardening of neck soft tissue. These can affect the blood supply of these structures, reducing the blood supply to the brain [16–18]. The bilateral temporal lobe blood supply is provided by the anterior branch of a superficial temporal artery which is separated by the bilateral middle cerebral artery and posterior cerebral artery. Hypertension and carotid/cerebral arteriosclerosis lead to vascular degeneration, affecting normal blood supply and the metabolism of these regions and increasing the risk of radiation-induced encephalopathy [19].

Elderly patients with lower physical function, lower immunity, and many underlying diseases may have softer-than-normal tissues that are more sensitive to ray irradiation, leading to an increased incidence rate of radiation-induced encephalopathy [20]. In clinically late-stage patients, lesions may appear in a wide range of locations, including the skull base, pterygopalatine fossa, anterior and posterior cranial nerves, cavernous sinus, and infratemporal fossa, so the radiation field and the exposed brain tissues need to be correspondingly expanded. Therefore, the incidence of radiation-induced encephalopathy is higher than in the early stages. For advanced NPC patients, we should use advanced radiotherapy to reduce the volume of exposed brain tissues and the exposure dose. Previous studies have shown that the higher the total radiation dose is, the higher the split dose, and the greater the incidence of radiation-induced encephalopathy [21]. In clinical treatment in China, the primary foci dose is often 70 Gy. However, there are still many patients who acquire radiation-induced encephalopathy, and thus the radiation dose is not the only important issue, and other factors may account for a large proportion of radiation-induced encephalopathy cases.

In this study, gender, multidrug combination chemotherapy, lymph node condition, diabetes mellitus, and metastasis showed no significant association with the occurrence of radiation-induced encephalopathy. In clinical treatment, induced chemotherapy, concurrent radiochemotherapy, or adjuvant chemotherapy can be combined with radiotherapy, and this is the key to increasing the control effective rate. Some research indicates that the application of combination chemotherapy increases the risk to normal soft tissues to a large extent [22]. Chemicals related to the cell cycle (such as methotrexate pteridine, mitomycin C, vincristine, and cyclophosphamide) can interact with radiation within rapid proliferation organization, thus significantly reducing brain tissue tolerance and increasing the radiation damage to brain tissue. However, our results were not consistent with these findings. Therefore, more research regarding the association between the occurrence of radiation-induced encephalopathy and multidrug combination chemotherapy is needed. In our next study, we may investigate the different chemotherapy regimens regarding their impact on the occurrence of radiation-induced encephalopathy. Previous literature also suggests that diabetes may contribute to radiation-induced encephalopathy [23]. The possible reason for this may be that hyperglycemia leads to macrovascular and microvascular degeneration, blood flow obstruction, and tissue hypoxia, and further aggravates the damage to blood–brain barrier function. However, few clinical studies have confirmed this speculation, and our study showed no significant association with diabetes. In addition, the number of cases with diabetes in our study was not large enough and this should be studied in a larger sample.

## 5 Clinical significance of this study

From the above analysis, we should focus more on NPC adults with older age, advanced disease, conventional therapy, hypertension or carotid/cerebral arteriosclerosis, and several courses of radiotherapy. The underlying disease of radiation-induced encephalopathy after NPC therapy should be actively cured, and reirradiation should be strictly controlled. Also, it is very crucial to explore novel biomarkers and targeted drugs in the NPC treatment [24,25], which can improve the NPC prognosis, to reduce the incidence of radiation-induced encephalopathy after NPC therapies.

## 6 Conclusion

In conclusion, radiation-induced encephalopathy of NPC patients should be examined with medical imaging rather than clinical examinations, and these patients should be followed up early and regularly, rather than only being treated after clinical symptoms appear. Radiotherapy method, radiation course, hypertension, carotid/cerebral arteriosclerosis, age, and clinical stage are found to be the riskiest factors associated with radiation-induced encephalopathy of NPC. We should intensify follow-up visits. In future studies, we will further explore how to prevent radiation-induced encephalopathy and protective measures for radiation-induced encephalopathy.
